# PET imaging of beta-secretase 1 in the human brain: radiation dosimetry, quantification, and test-retest examination of [^18^F]PF-06684511

**DOI:** 10.1007/s00259-020-04739-5

**Published:** 2020-03-06

**Authors:** Ryosuke Arakawa, Akihiro Takano, Per Stenkrona, Vladimir Stepanov, Sangram Nag, Mahabuba Jahan, Per Grybäck, Martin Bolin, Laigao Chen, Lei Zhang, Ping He, Anabella Villalobos, Timothy J. McCarthy, Christer Halldin, Andrea Varrone

**Affiliations:** 1 Centre for Psychiatry Research, Department of Clinical Neuroscience, Karolinska Institutet, & Stockholm Health Care Services, Region Stockholm, Stockholm, Sweden; 2 Medical Radiation Physics and Nuclear Medicine, Karolinska University Hospital, Stockholm, Sweden; 3 Worldwide Research & Development, Pfizer Inc., Cambridge, MA USA

**Keywords:** Beta-secretase 1 (BACE1), Dosimetry, Human brain, Positron emission tomography (PET), Test-retest repeatability

## Abstract

**Purpose:**

Beta-secretase 1 (BACE1) enzyme is implicated in the pathophysiology of Alzheimer’s disease. [^18^F]PF-06684511 is a positron emission tomography (PET) radioligand for imaging BACE1. Despite favorable brain kinetic properties, the effective dose (ED) of [^18^F]PF-06684511 estimated in non-human primates was relatively high. This study was therefore designed to evaluate the whole-body distribution, dosimetry, quantification, and test-retest reliability of imaging brain BACE1 with [^18^F]PF-06684511 in healthy volunteers.

**Methods:**

Five subjects were studied for the dosimetry study. Whole-body PET was performed for 366 min with 4 PET-CT sessions. Estimates of the absorbed radiation dose were calculated using the male adult model. Eight subjects participated in the test-retest study. Brain PET measurements were conducted for 123 min with an interval of 5 to 19 days between test and retest conditions. The total distribution volume (*V*_T_) was estimated with one-tissue (1T), two-tissue (2T), compartment model (CM), and graphical analysis. Test-retest variability (TRV) and intraclass correlation coefficient (ICC) of *V*_T_ were calculated as reliability measures.

**Results:**

In the dosimetry study, the highest uptake was found in the liver (25.2 ± 2.3 %ID at 0.5 h) and the largest dose was observed in the pancreas (92.9 ± 52.2 μSv/MBq). The calculated ED was 24.7 ± 0.8 μSv/MBq. In the test-retest study, 2TCM described the time-activity curves well. *V*_T_ (2TCM) was the highest in the anterior cingulate cortex (6.28 ± 1.09 and 6.85 ± 0.81) and the lowest in the cerebellum (4.23 ± 0.88 and 4.20 ± 0.75). Mean TRV and ICC of *V*_T_ (2TCM) were 16.5% (12.4–20.5%) and 0.496 (0.291–0.644).

**Conclusion:**

The ED of [^18^F]PF-06684511 was similar to other ^18^F radioligands, allowing repeated PET measurements. 2TCM was the most appropriate quantification method. TRV of *V*_T_ was similar to other radioligands without a reference region, albeit with lower ICC. These data indicated that [^18^F]PF-06684511 is a suitable radioligand to measure BACE1 level in the human brain.

**Trial registration:**

EudraCT 2016-001110-19 (registered 2016-08-08)

## Introduction

Beta-secretase 1 (beta-site amyloid precursor protein (APP)–cleaving enzyme 1; BACE1) enzymatic activity is implicated in the pathophysiology of brain amyloid-beta (Aβ) accumulation in Alzheimer’s disease (AD). BACE1 is an enzyme responsible for the cleavage of APP, a process which represents the first step in the amyloidogenic pathway leading to the formation of Aβ [1]. BACE1 inhibitors have been considered potential agents for the treatment of AD by reducing the production of Aβ [2, 3]. A positron emission tomography (PET) imaging agent for the quantification of BACE1 activity in the brain can be an important tool to complement the existing portfolio of radiopharmaceuticals used for evaluation of patients with cognitive impairment in neurodegenerative diseases such as AD.

The PET radioligand [^18^F]PF-06684511 has been developed for in vivo imaging and quantification of BACE1 [4]. Recently, we showed that [^18^F]PF-06684511 displays suitable kinetic properties in the brain of non-human primates (NHPs) and also reported a dose-dependent blocking effect on [^18^F]PF-06684511 brain uptake by BACE1 inhibitors [5]. These data suggested that [^18^F]PF-06684511 is a promising PET radioligand for brain imaging of BACE1. In addition to brain imaging, we also evaluated the whole-body distribution and dosimetry of [^18^F]PF-06684511 in NHPs [5]. Remarkably, we found that the effective dose (ED) in humans estimated from NHP data was 43 μSv/MBq, a value that is relatively high compared with the ED of other ^18^F radioligands [6]. This relatively high ED was related to high uptake of [^18^F]PF-06684511 in the gastric lumen. The reasons for the high stomach uptake are not known, but the experimental setting such as the usage of anesthesia and the recumbent position of the NHP for long time might have played a role.

Altogether, the results obtained in NHPs suggested that [^18^F]PF-06684511 could be a suitable PET radioligand for brain imaging of BACE1. On the other hand, the results also suggested that further studies were needed to examine the dosimetry, the method of quantification, and the reliability of [^18^F]PF-06684511 in human subjects.

The aims of the present work were therefore (1) to calculate the radiation dose from the administration of [^18^F]PF-06684511 in healthy volunteers based on whole-body PET/CT scans (study A) and (2) to examine the quantification and test-retest reliability of [^18^F]PF-06684511 binding to BACE1 in the brain of healthy volunteers (study B).

## Methods

### Subjects

Five healthy subjects (3 females and 2 males, 41–53 years) for study A (dosimetry) and eleven healthy subjects (9 females, 2 males, 41–59 years) for study B (test-retest) were included. The subjects gave oral and written informed consent to participate in the study. They were considered healthy on the basis of medical and psychiatric history, physical examination, drug screening, ECG, and blood tests. In study B, subjects were also required to have an unremarkable magnetic resonance imaging (MRI) scan prior to inclusion. The study was approved by the Swedish Medicinal Products agency and the ethical committee of the Stockholm region. The use of [^18^F]PF-06684511 was approved by the Radiation Safety Committee of the Karolinska University Hospital. The study was registered to the EudraCT (2016-001110-19).

### Radioligand synthesis

[^18^F]PF-06684511 was synthesized as reported previously [4, 5]. In brief, an aqueous [^18^F]fluoride was produced via the ^18^O(p,n)^18^F nuclear reaction using a GE Medical Systems PETtrace cyclotron in a silver fluoride-18 water target. The radionuclide was transferred from the target to the synthesis module (custom ^18^F-module by Scansys, Denmark) by means of helium flow (in a 1.5-ml bolus of [^18^O]H_2_O) and trapped on a QMA light Sep-Pak cartridge (bicarbonate form) to remove [^18^O]H_2_O. [^18^F]fluoride was then eluted into the reaction vessel using 1.5 ml of acetonitrile/water (96/4 v/v) containing 7.4 mg of Kryptofix 2.2.2 and 1.4 mg of potassium carbonate. The solvents were evaporated by heating at 140 °C under a stream of nitrogen (100 ml/min). To the dried [^18^F]fluoride, complex 1.6–2.4 mg of precursor (PF-06816649, Pfizer) dissolved in 500 μl of DMSO was added. The reaction mixture was heated for 5 min at 130 °C without stirring. The removal of the Boc-protecting group was subsequently carried out by adding 500 μl of trifluoroacetic acid-water (1:1) to the reaction mixture and heating at 90 °C for 5 min. The resulting mixture was diluted with 3.3 M NaOH to slightly acidic pH, injected onto ACE 5 C18-HL HPLC column (250 × 10 mm), and eluted with mixture of MeCN/0.1% aq. TFA (250:750). The labeled product fraction was collected and diluted in 50 ml of sterile water. It was then concentrated on a Sep-Pak Plus tC18 Short cartridge, wasted with 8 mL of sterile water, and eluted from cartridge with ca 1.5 ml of 99.5% ethanol into ca. 14 ml of sterile phosphate-buffered saline. The resulting formulation was then filtered through a Millex-GV 0.22 μm sterile filter, and samples for QC were taken after filtration.

Formulated product was analyzed for compliance with the GMP criteria approved by the Swedish Medical Products Agency after prior submission of validation results and CMC documentation: radiochemical purity, molar activity, amount of UV-adsorbing impurities, and residual Kryptofix were determined during QC analysis. On average, 200 to 800 MBq of [^18^F]PF-06684511 was produced (*n* = 11) (1–5% RCY, decay corrected). Radiochemical purity was ≥ 95% for all production runs. Molar radioactivity fell in the range of 33 to 77 GBq/μmol for all batches of [^18^F]PF-06684511 produced.

### Study A (dosimetry)

#### PET and CT measurement

PET measurements were performed with a GE Discovery PET/CT 710 (GE healthcare, Waukesha, WI, USA). The radioligand [^18^F]PF-06684511 was administered intravenously as a bolus injection within 10 s. Four PET sessions with breaks in between were performed as follows to cover the subject’s body from head to upper thigh: (1) 2 min × 9 bed positions × 3 times (0–54 min), (2) 3 min × 9 bed positions × 2 times (90–144 min), (3) 3 min × 9 bed positions × 2 times (210–264 min), and (4) 4 min × 9 bed positions × 1 time (330–366 min). One low-dose CT scan was performed before each session for attenuation correction. To calculate the radioactivity in the urine, urine samples were collected and measured with a PET/CT system with 5-min acquisitions at each break.

#### Image analysis and radiation dose estimation

PET images were reconstructed with a 3D ordered subset expectation maximization (OSEM) algorithm with three iterations and eighteen subsets, including the time of flight information (VUE point FX) and the point spread function correction (sharp IR). Volumes of interests (VOIs) were defined on fused PET and CT images acquired during the 1st session. VOIs were delineated on the brain, salivary glands, thyroid, lung, heart, liver, pancreas, gall bladder, spleen, stomach, kidney, small intestine, lumbar vertebrae, and urinary bladder. The CT images from the 2nd to the 4th sessions were co-registered to CT images of the 1st session. The co-registration parameters were applied to PET images of corresponding sessions. The same VOIs were applied to calculate the time activity curves (TACs). TACs were corrected for decay and the uptake in each organ was reported as %injected dose (%ID), where %ID = radioactivity (Bq/cc) × VOI volume (cc) / injected dose (Bq) × 100. To calculate the %ID in the urinary bladder, the radioactivity measured in the urine samples was added to that measured in VOI delineated on the urinary bladder. Values of %ID per organ were fit using the SAAM II software. Estimates of the absorbed radiation dose were calculated with the OLINDA/EXM software using the male adult model [7].

### Study B (test-retest)

#### MRI and PET measurements

After health screening and before enrollment in the study, each subject underwent a T2-weighted and a T1-weighted MRI scans for a total duration of approximately 30 min using a 1.5-T Siemens MAGNETOM Avanto. The purpose of the MRI was to rule out pathology and to help the anatomical delineation of brain VOIs.

After being enrolled into the study, each subject underwent two PET measurements within an interval of 5 to 19 days. Due to technical reasons, PET measurements were performed in the first five subjects using an ECAT EXACT HR system (Siemens Molecular Imaging, Knoxville, TN) and in the remaining three subjects using a HRRT system (Siemens Molecular Imaging, Knoxville, TN). A plaster helmet was made for each subject and used with a head fixation system during the PET measurements. A transmission scan for attenuation correction was acquired prior to the emission scan for approximately 10 min with three rotating ^68^Ge sources for the ECAT EXACT HR, and for approximately 6 min with a single ^137^Cs source for the HRRT. The radioligand [^18^F]PF-06684511 was administered intravenously as a bolus injection within 10 s. PET measurements conducted with the ECAT EXACT HR system were acquired in 3D mode for 123 min, using a series of 34 frames of increasing durations (20 s × 9, 1 min × 3, 3 min × 5, 6 min × 17). PET measurements conducted with the HRRT system were acquired in list mode for 123 min and subsequently reconstructed with the same number of frames and durations as in the case of the ECAT EXACT HR system.

### Arterial blood sampling

Arterial blood was collected continuously for 5 min using an automated blood sampling system (Twilite, Swisstrace, Switzerland) at a speed of 5 ml/min and then collected manually up to the end of the PET measurement. Radioactivities in whole blood and plasma of arterial blood were measured at 1, 2, 3, 4, 5, 6, 8, 10, 20, 30, 45, 60, 90, and 120 min after the PET radioligand injection.

### Radiometabolite analysis

Arterial blood samples were collected at 2, 5, 10, 20, 30, 45, 60, 90, and 120 min after [^18^F]PF-06684511 injection for radiometabolite analysis to determine the percentage of unchanged radioligand and radiometabolites in the human plasma. The plasma (0.7–1.5 ml) obtained after centrifugation of whole blood (2–4 ml) at 2000*g* for 2 min was mixed with acetonitrile (using a volume 1.4 times larger than the plasma) to precipitate proteins in the plasma. After stirring with a vortex mixer, the sample was centrifuged for 4 min at 2000*g* and the supernatant plasma-acetonitrile mixture was separated from the protein precipitate. The protein precipitate was washed with extra acetonitrile. In order to improve the peak shape, the supernatant was diluted with 2–3 ml of water before being injected into a high-performance liquid chromatography (HPLC). The recovery of the sample preparation was determined by measuring the radioactivity of the plasma, supernatant and protein precipitate in a NaI well-counter.

A HPLC method was developed to separate radiometabolites from the parent radioligand [^18^F]PF-06684511. The radio-HPLC system used for radiometabolite analysis consisted of an interface module (D-7000, Hitachi), a pump (L-7100, Hitachi), and an injector (7125, 5.0 ml loop, Rheodyne, Cotati, CA, USA). A 5-ml sample was injected into the semi-preparative reverse phase (RP) C18 ACE column (2.5 μm, 50 × 10 mm) equipped with an UV absorbance detector (L-7100, wavelength 254 nm; Hitachi) in series with a 150TR Packard radiodetector (550-ml flow cell) for radioactivity determination.

A gradient method was used with a flow rate of 5 ml/min using acetonitrile (A) and 10 mM ammonium formate (B) as the mobile phase (gradient method 0–3.5 min, (A/B) 50:50 v/v-70:30 v/v → 3.5–4.5 min, 70:30–80:20 v/v; 4.5–6.0 min, 80:20 v/v; 6.1–8.0 min 50:50 v/v). The percentage of the unchanged radioligand was calculated as (integrated peak area corresponding to parent radioligand / sum of the integrated peak areas of all detected radiometabolites) × 100.

### Protein binding

Plasma (500 μl) or phosphate-buffered saline solution (500 μl) as a control was mixed with the formulation (50 μl, ~ 1–5 MBq) and incubated at room temperature for 10 min. After the incubation, 200-μl portions of the incubation mixtures was pipetted into ultrafiltration tubes (Centrifree YM-30, molecular weight cutoff, 30,000; Millipore: Billerica, USA) and centrifuged at 1500*g* for 15 min. Equal aliquots (20 μl) of the ultrafiltrate (C_free_) and of the plasma (C_total_) were counted for their radioactivity using a NaI well counter. Each determination was performed in duplicate. The free fraction was then calculated as *f*_P_=C_free_/C_total_, and the results were corrected for the membrane binding measured with the control samples.

### Image reconstruction and image processing

For the ECAT EXACT HR, images were reconstructed with 2D filtered back projection using a Hanning filter (2.0 mm) [8]. For the HRRT, images were reconstructed with the ordinary Poisson 3D ordered subset expectation maximization (OP-3D-OSEM) algorithm with 10 iterations and 16 subsets including modeling of the point spread function (PSF) [9]. The MR image was segmented into gray matter (GM), white matter (WM), and cerebrospinal fluid (CSF) images using the SPM5 segmentation algorithm in MATLAB (Wellcome Trust Centre for Neuroimaging, London, UK; The MathWorks, Inc., Natick, MA, USA). VOIs were defined as cerebellar cortex (CERCX), caudate (CAU), putamen (PUT), thalamus (THA), lateral frontal cortex (LFC), lateral temporal cortex (LTC), hippocampus (HIP), lateral occipital cortex (LOC), lateral parietal cortex (LPC), anterior cingulate cortex (ACC), posterior cingulate cortex (PCC), and amygdala (AMG) using the anatomical automatic labeling (AAL) template. GM masking was applied to the AAL template. All MRIs and VOIs were co-registered to summated PET images with the mutual information algorithm using SPM5. Regional TACs were calculated for each frame, corrected for decay, and plotted vs. time.

### Kinetic model analysis

The main outcome measure was the total distribution volume (*V*_T_) estimated using one-tissue compartment model (1-TCM, *V*_T_ = *K*_1_ / *k*_2_), two-tissue compartment model (2-TCM, *V*_T_ = (*K*_1_ / *k*_2_) × (*k*_3_ / *k*_4_ + 1)), and Logan graphical analysis (GA) [10] with metabolite-corrected arterial input function. Kinetic analysis was conducted using PMOD3.6 (PMOD Technologies Ltd., Zurich, Switzerland). The Akaike information criterion (AIC) and the F-test were used to assess whether the 2-TCM significantly improved the fitting of the data as compared with the 1-TCM.

### Time stability

To check the time stability of *V*_T_ of GM by 2-TCM, the PET data was truncated to 63 min in 12-min steps. The data is presented as the %difference of *V*_T_ to that of the whole 123 min.

### Test-retest repeatability and intraclass correlation coefficient

The reliability of *V*_T_ was calculated using the test-retest variability (TRV) as a measure of repeatability and the intraclass correlation coefficient (ICC). The TRV was expressed by the absolute difference of the values between the first and second PET measurements (PET1 and PET2) relative to the mean of the two values according to the following equation: TRV(%) = |PET2 − PET1| / ((PET1 + PET2) / 2) × 100.

The ICC was measured according to the following equation: ICC = (s_b_^2^ − s_w_^2^) / (s_b_^2^ + (*n*−1) × s_w_^2^), where s_b_^2^ = mean sum of square between subjects, s_w_^2^ = mean sum of square within subjects, and *n* = number of within-subject measurements (*n* = 2 in this study).

To investigate the possible sources of variability of the *V*_T_ data, TRV and ICC were also calculated for the area under the curve (AUC) of the GM TACs, the AUC of the arterial plasma input function, and the ratio of AUC GM / AUC plasma input. To investigate the reliability of the measurement of protein binding, TRV and ICC of *f*_P_ were also calculated.

## Results

Five subjects were included in study A and 11 subjects were included in study B. In study B, two subjects performed only the first PET measurement. In one subject, for technical reasons, the data collected with the blood sampling system from both PET measurements were not available. Eight subjects completed the study according to the protocol. No adverse or pharmacological effects of [^18^F]PF-06684511 were observed in any of the participants. Two participants reported headache, one participant reported back pain, and one participant reported symptoms of common cold and showed high systolic blood pressure. Two subjects had a small hematoma on the site of arterial cannulation. All these adverse events were considered mild and probably related to the PET procedure.

### Study A (dosimetry)

The injected radioactivity (*n* = 5) of [^18^F]PF-06684511 was 111 ± 13 (mean ± SD) (range, 93–130) MBq. The molar radioactivity at the time of injection was 42 ± 11 (34–59) GBq/μmol, and the injected mass was 1.19 ± 0.25 (0.88–1.38) μg.

**Fig. 1:**
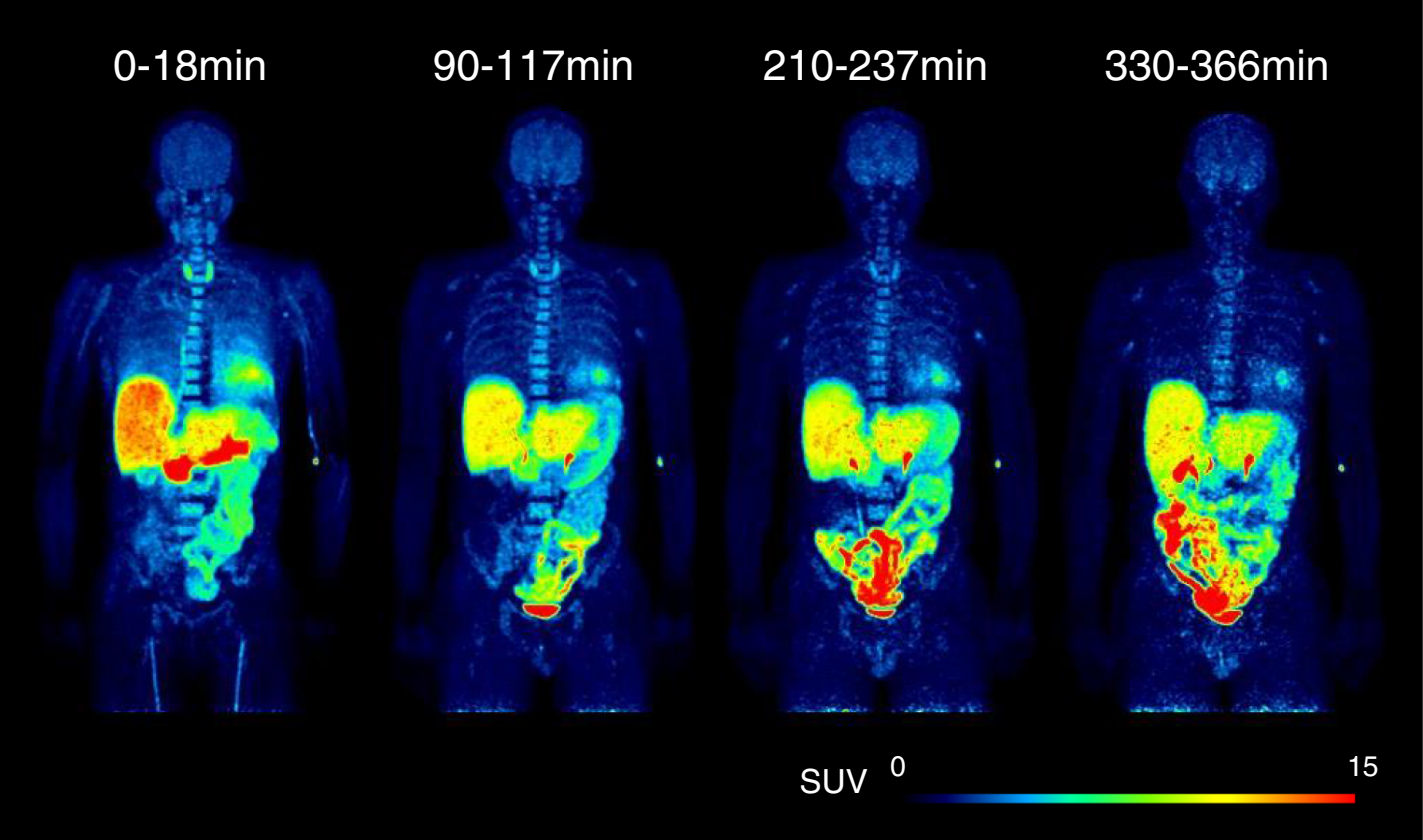
Representative whole body PET images at different times. Images are presented by maximum intensity projection (MIP)

**Fig. 2:**
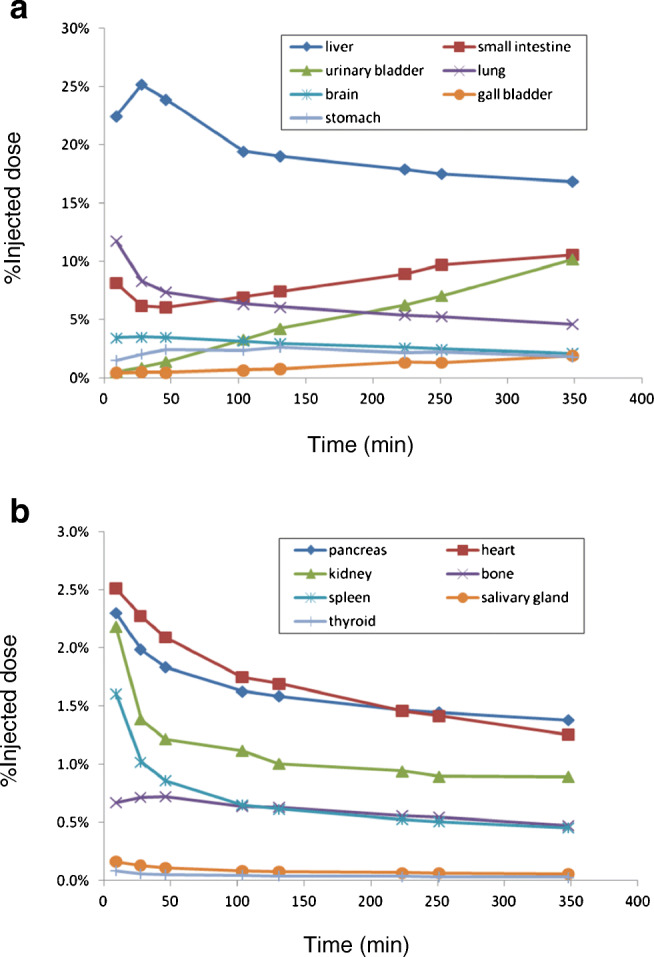
Mean time activity curves of [^18^F]PF-06684511 in different organs for high uptakes (**a**) and low uptakes (**b**)

Whole body PET maximum intensity projection (MIP) images of one representative subject at different time points after injection are shown in Fig. 1. Mean time activity curves expressed as %ID are shown in Fig. 2a, b. Peak uptake values were the highest in the liver (25.2 ± 2.3 %ID at 0.5 h) and lungs (11.8 ± 2.9 %ID at 0.2 h), followed by the small intestine (10.6 ± 3.1 %ID at 5.8 h), brain (3.5 ± 0.6 %ID at 0.5 h), stomach (2.6 ± 1.0 %ID at 2.2 h), heart (2.5 ± 0.5 %ID at 0.2 h), and pancreas and kidneys (2.3 ± 0.7 %ID and 2.2 ± 1.0 %ID at 0.2 h). Up to 10.2 ± 4.1 %ID was eliminated with the urine at 5.8 h.

The human radiation dose estimates are shown in Table 1. The organ receiving the largest dose was the pancreas (92.9 ± 52.2 μSv/MBq), followed by the liver (70.5 ± 5.9 μSv/MBq), the gall bladder and urinary bladder wall (57.7 ± 40.9 and 57.5 ± 21.2 μSv/MBq), and the ULI wall (51 ± 11.1 μSv/MBq). The calculated ED was 24.7 ± 0.8 μSv/MBq.

**Table 1:** Human radiation doses estimates of [^18^F]PF-06684511

	Mean	SD
Adrenals	16.8	1.0
Brain	15.6	0.9
Breasts	7.66	0.3
Gallbladder wall	57.7	40.9
LLI wall	21.7	3.4
Small intestine	45.5	9.8
Stomach wall	13.1	0.9
ULI wall	51	11.1
Heart wall	35.7	6.5
Kidneys	29.4	4.7
Liver	70.5	5.9
Lungs	37.1	2.4
Muscle	9.28	0.2
Ovaries	15.2	1.4
Pancreas	92.9	52.2
Red marrow	18.6	1.2
Osteogenic cells	40	3.5
Skin	6.37	0.2
Spleen	28.8	10.0
Testes	7.26	0.4
Thymus	9.28	0.5
Thyroid	14.9	4.7
Urinary bladder wall	57.5	21.2
Uterus	15.2	1.2
Total body	13.4	0.2
Effective dose	24.7	0.8

Unit: μSv/MBq

### Study B (test-retest)

#### PET acquisition

**Fig. 3:**
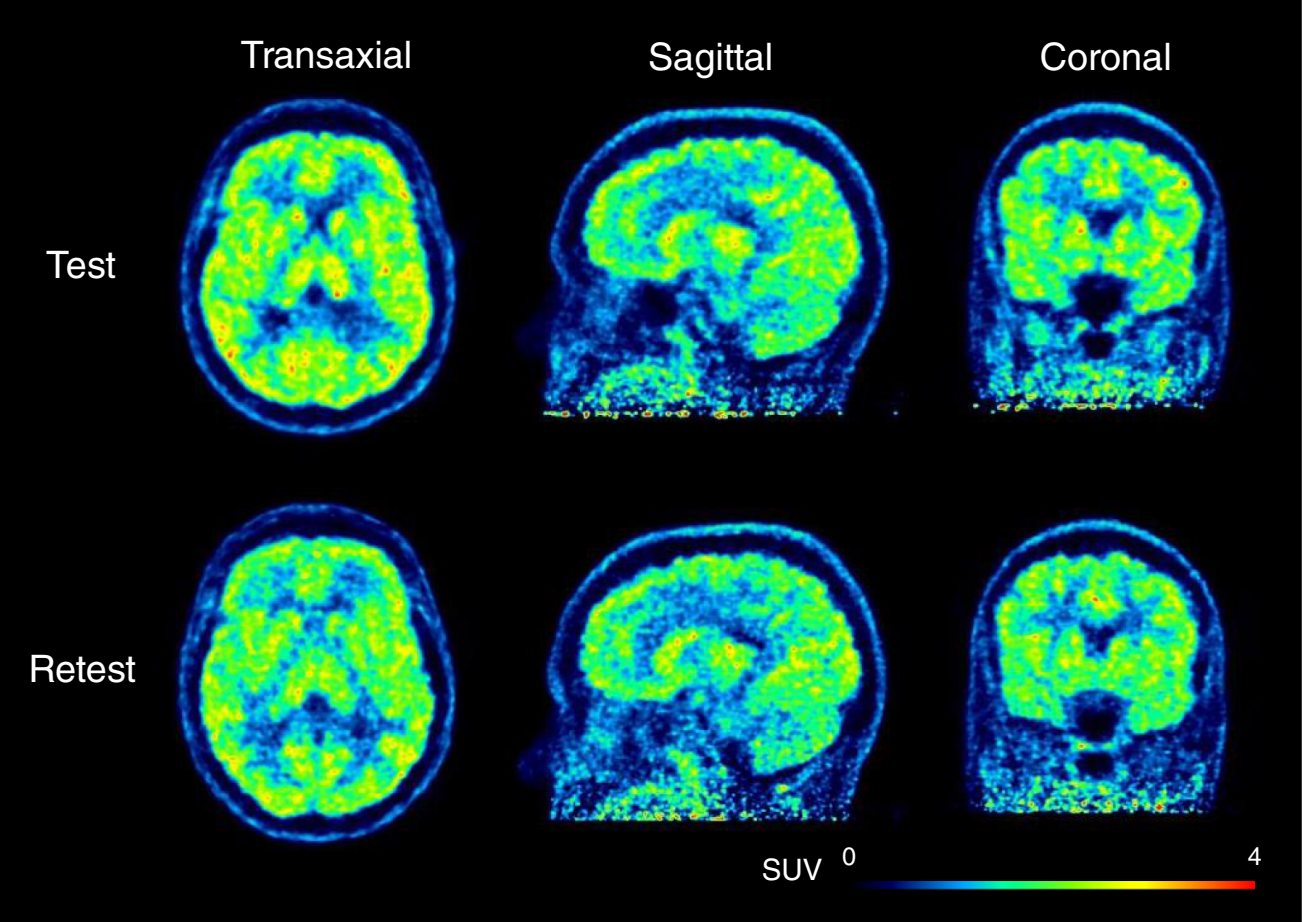
Representative summated brain PET images (0–123 min)

The amounts of radioactivity ([^18^F]PF-06684511) administered for test and retest portions were 122 ± 22 (102–173) MBq and 122 ± 22 (103–162) MBq, respectively. The molar radioactivity at the time of injection for each portion was 54 ± 14 (33–77) GBq/μmol and 58 ± 10 (44–72) GBq/μmol, respectively. The injected masses were 1.06 ± 0.40 (0.58–1.71) μg and 0.95 ± 0.29 (0.70–1.53) μg, respectively. Summated PET images of one representative subject are shown in Fig. 3. [^18^F]PF-06684511 distributed rather uniformly in the brain gray matter.

#### Time activity curve in the brain

**Fig. 4:**
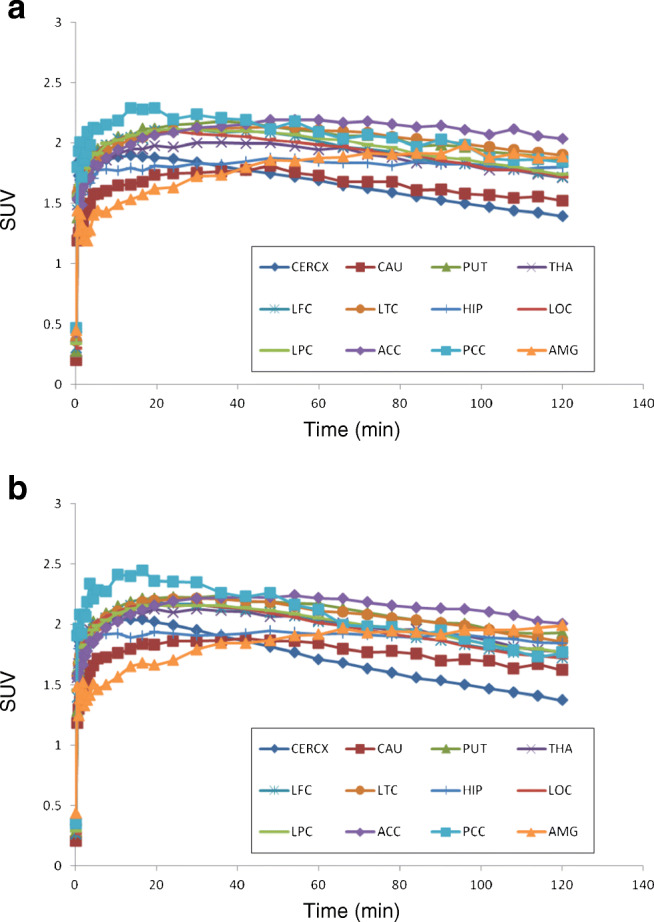
Mean time activity curves of [^18^F]PF-06684511 in different brain regions for test (**a**) and retest (**b**) measurement. CERCX, cerebellum; CAU, caudate; PUT, putamen; THA, thalamus; LFC, frontal cortex; LTC, temporal cortex; HIP, hippocampus; LOC, occipital cortex; LPC, parietal cortex; ACC, anterior cingulate cortex; PCC, posterior cingulate cortex; AMG, amygdala

The mean whole brain uptake was peaked around 1.8 standardized uptake value (SUV) at approximately 30 min after injection with relatively slow washout (mean SUV 1.6 at 120 min). Mean regional brain TACs of the test and retest PET measurements are shown in Fig. 4a, b. The highest brain uptake was in the anterior cingulate cortex, and the lowest was in the cerebellum at the later time points.

#### Plasma metabolite analysis and protein binding

**Fig. 5:**
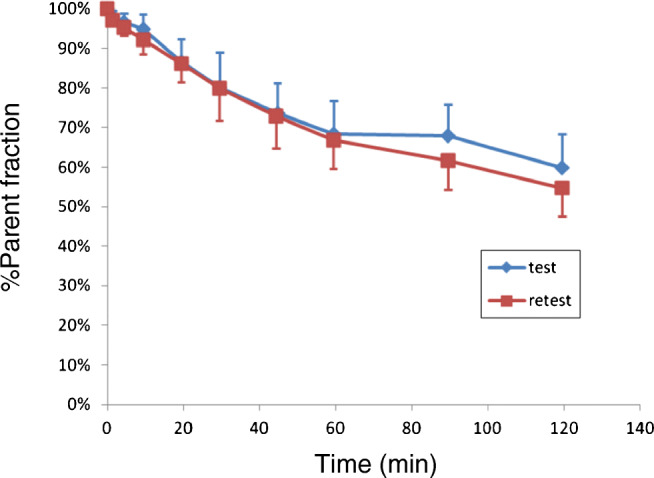
Mean parent fraction curves of [^18^F]PF-06684511. Values are mean + 1SD of all subjects

**Fig. 6:**
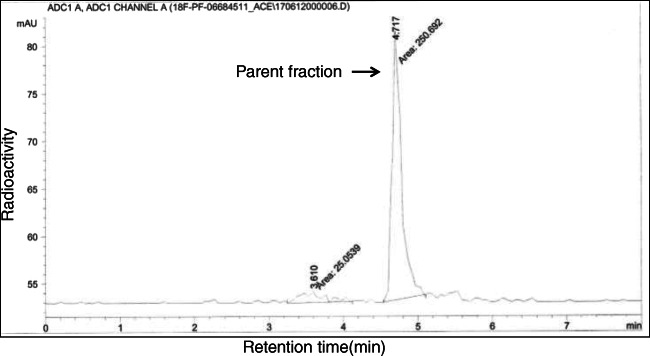
Representative radio-HPLC

The mean percent values of plasma parent fraction are shown in Fig. 5. [^18^F]PF-06684511 in the plasma was rather stable, with 70% of the unchanged radioligand still present at 60 min after injection. All detected radiometabolites were more polar than the parent radioligand (Fig. 6). The mean *f*_P_ was 9.3 ± 2.5% and 9.8 ± 2.6% for test and retest measurements, respectively.

#### Kinetic parameter and total distribution volume (*V*_T_)

**Fig. 7:**
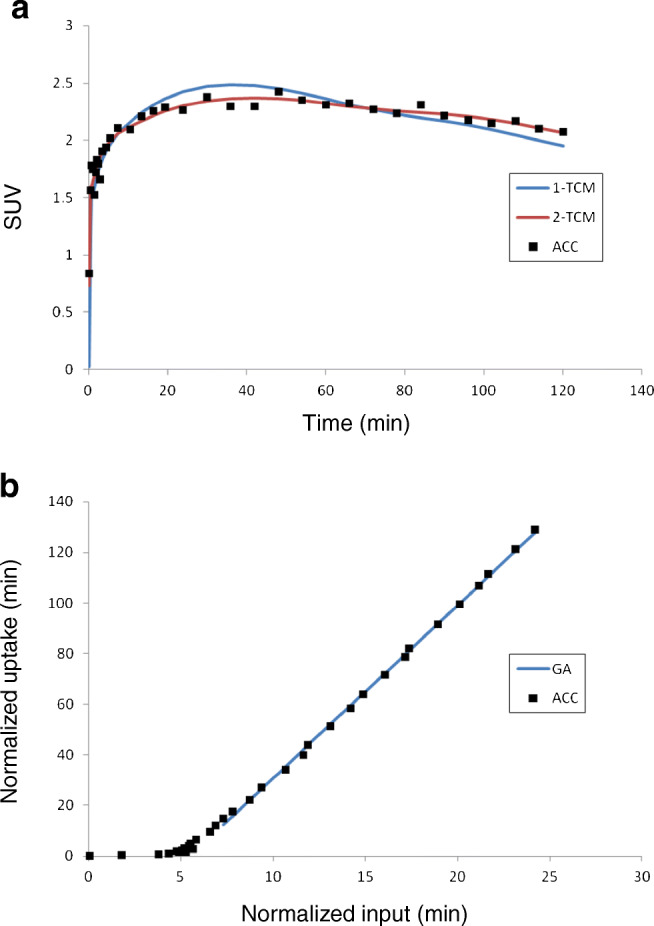
Representative fitting curves of 1-TCM and 2-TCM (**a**) and GA (**b**) in anterior cingulate cortex (ACC)

**Fig. 8:**
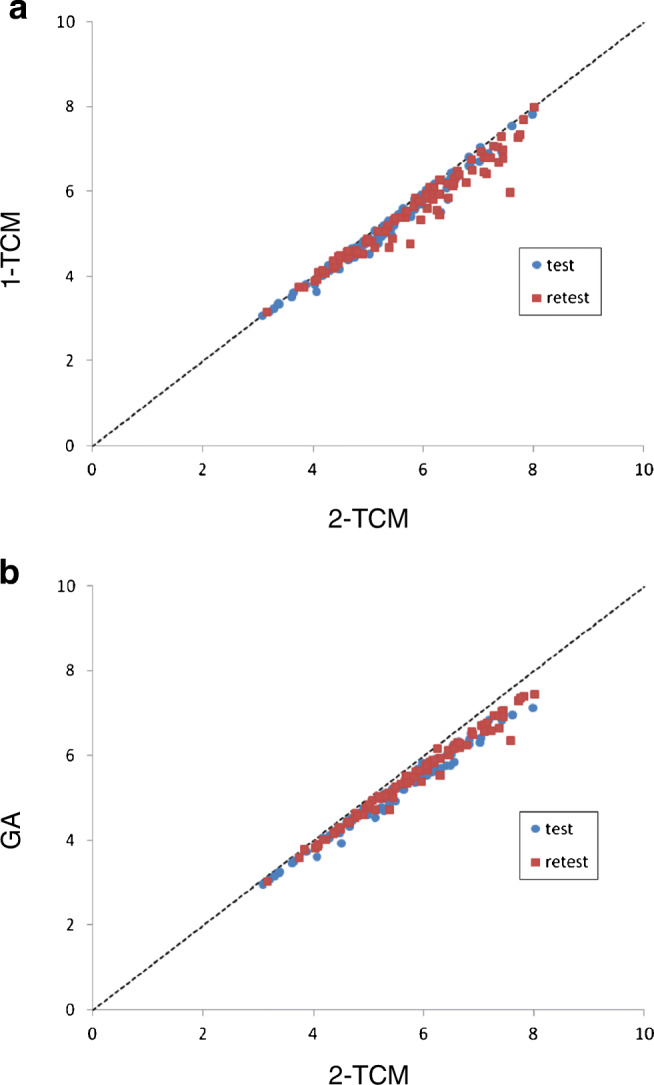
Scatter plot of V_T_ by 2-TCM vs. 1-TCM (**a**) and GA (**b**). **a** Test: *Y* = 0.97*X R*^2^ = 0.98, retest: *Y* = 0.95*X R*^2^ = 0.95. **b** Test: *Y* = 0.94*X R*^2^ = 0.98, retest: *Y* = 0.94*X R*^2^ = 0.98

Representative fitting curves of 1-TCM, 2-TCM, and GA in anterior cingulate cortex are shown in Fig. 7a, b. Kinetic parameters *V*_T_ using 1-TCM, 2-TCM, and GA are shown in Table 2. Two data, putamen of one subject and anterior cingulate cortex of another subject, by 2-TCM were excluded because of no convergence. The highest *V*_T_ values using 2-TCM were in the anterior cingulate cortex (test, 6.28 ± 1.09; retest, 6.85 ± 0.81) and amygdala (6.00 ± 1.08, 6.73 ± 1.25) and the lowest were in the caudate (4.56 ± 0.82, 5.29 ± 0.87) and the cerebellum (4.23 ± 0.88, 4.50 ± 0.75). The AIC of 2-TCM was lower than that of 1-TCM in 89% and 86% fitting curves for test and retest measurement, respectively. Also, *F*-test showed 2-TCM was better than 1-TCM (*p* < 0.05) in 88% and 85% fitting curves for test and retest measurement, respectively. The *V*_T_ values by 1-TCM and GA were well correlated to those by 2-TCM with slightly underestimation as around 4% and 6%, respectively (Fig. 8a, b).

**Table 2:** Kinetic parameters and *V*_T_ by 1-TCM, 2-TCM, and GA for test and retest measurement

	1-TCM						2-TCM										GA	
	*K*_1_		*k*_2_		*V*_T_		*K*_1_		*k*_2_		*k*_3_		*k*_4_		*V*_T_		*V*_T_	
	mean	SD	mean	SD	mean	SD	mean	SD	mean	SD	mean	SD	mean	SD	mean	SD	mean	SD
Test																		
CERCX	0.128	0.041	0.031	0.006	4.12	0.88	0.202	0.067	0.747	0.794	0.718	0.283	0.085	0.053	4.23	0.88	4.02	0.78
CAU	0.103	0.033	0.023	0.005	4.45	0.79	0.116	0.038	0.149	0.134	0.561	0.605	0.170	0.186	4.56	0.82	4.28	0.74
PUT	0.129	0.040	0.024	0.006	5.46	1.03	0.159	0.042	0.214	0.131	0.625	0.356	0.129	0.147	5.78	0.91	5.25	0.88
THA	0.123	0.040	0.024	0.004	4.97	0.86	0.157	0.042	0.263	0.202	0.454	0.299	0.079	0.044	5.13	0.88	4.84	0.81
LFC	0.130	0.050	0.025	0.006	5.06	0.98	0.154	0.054	0.242	0.265	0.595	0.516	0.168	0.197	5.16	1.01	4.86	0.92
LTC	0.125	0.043	0.022	0.005	5.52	0.93	0.163	0.040	0.371	0.316	0.629	0.340	0.087	0.080	5.67	0.95	5.35	0.82
HIP	0.115	0.038	0.023	0.006	4.89	0.92	0.162	0.048	0.320	0.328	0.304	0.249	0.038	0.014	5.28	0.93	4.90	0.88
LOC	0.133	0.053	0.026	0.007	5.01	0.94	0.167	0.060	0.289	0.275	0.518	0.298	0.110	0.096	5.11	0.95	4.84	0.87
LPC	0.131	0.055	0.025	0.006	5.13	1.10	0.165	0.063	0.378	0.530	0.607	0.511	0.157	0.187	5.23	1.15	4.93	1.06
ACC	0.119	0.041	0.020	0.004	5.97	0.98	0.150	0.039	0.219	0.190	0.445	0.265	0.081	0.064	6.28	1.09	5.79	0.87
PCC	0.144	0.050	0.027	0.007	5.29	0.84	0.203	0.037	0.634	0.495	0.854	0.675	0.065	0.028	5.44	0.84	5.15	0.73
AMG	0.087	0.026	0.015	0.003	5.70	1.08	0.132	0.040	0.480	0.399	0.540	0.351	0.051	0.042	6.00	1.08	5.54	0.93
Retest																		
CERCX	0.138	0.029	0.032	0.009	4.38	0.71	0.233	0.094	0.693	0.613	0.560	0.269 0	794	2.076	4.50	0.75	4.33	0.71
CAU	0.107	0.021	0.022	0.006	5.07	0.83	0.136	0.051	0.287	0.607	0.236	0.321	0.718	1.681	5.29	0.87	4.98	0.78
PUT	0.131	0.026	0.023	0.006	5.92	1.05	0.158	0.055	0.149	0.244	0.201	0.219	0.153	0.238	6.35	1.10	5.85	1.03
THA	0.131	0.027	0.025	0.007	5.48	0.93	0.182	0.059	0.299	0.344	0.344	0.181	0.070	0.047	5.74	1.04	5.46	0.93
LFC	0.132	0.035	0.025	0.006	5.39	0.82	0.171	0.061	0.293	0.411	0.316	0.332	0.591	1.462	5.60	0.84	5.29	0.81
LTC	0.130	0.029	0.023	0.006	5.83	0.89	0.187	0.075	0.424	0.541	0.428	0.320	0.083	0.063	6.00	0.94	5.73	0.87
HIP	0.123	0.031	0.023	0.009	5.43	0.91	0.19	0.081	0.424	0.543	0.281	0.219	0.040	0.023	6.02	1.11	5.57	0.91
LOC	0.135	0.036	0.025	0.006	5.35	0.89	0.196	0.083	0.472	0.563	0.505	0.561	0.089	0.078	5.52	0.94	5.27	0.89
LPC	0.132	0.038	0.024	0.006	5.52	0.99	0.178	0.067	0.358	0.435	0.443	0.432	0.114	0.143	5.71	1.05	5.42	1.00
ACC	0.123	0.027	0.020	0.005	6.34	1.02	0.179	0.061	0.425	0.427	0.526	0.247	0.056	0.031	6.85	0.81	6.25	1.01
PCC	0.156	0.039	0.029	0.008	5.48	0.97	0.269	0.118	0.793	0.739	0.584	0.369	0.123	0.202	5.65	1.04	5.35	0.93
AMG	0.092	0.019	0.015	0.005	6.24	1.21	0.152	0.058	0.479	0.508	0.393	0.277	0.108	0.225	6.73	1.25	6.30	1.12

*CERCX*, cerebellum; *CAU*, caudate; *PUT*, putamen; *THA*, thalamus; *LFC*, frontal cortex; *LTC*, temporal cortex; *HIP*, hippocampus; *LOC*, occipital cortex; *LPC*, parietal cortex; *ACC*, anterior cingulate cortex; *PCC*, posterior cingulate cortex; *AMG*, amygdala

**Fig. 9:**
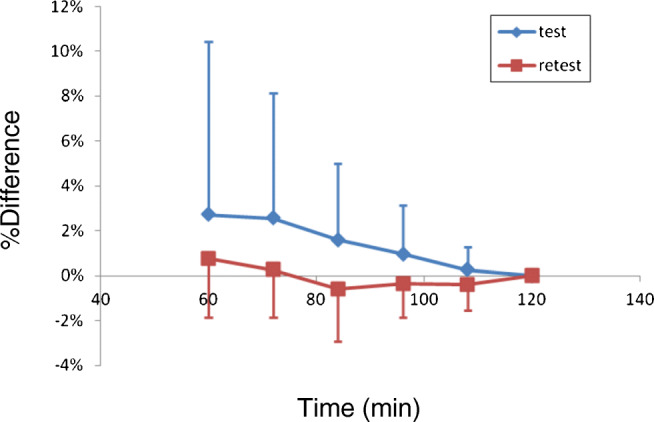
Time stability analysis of GM V_T._ Values are mean + 1SD of all subjects

The analysis of the time stability of GM *V*_T_ estimated with 2-TCM is shown in Fig. 9. With the shortest time of the image analysis (63 min), the mean GM *V*_T_ value was still within 5% difference of the value at 123 min. The SD of GM *V*_T_ was lower than 5% for data analyzed with the duration of at least 87 min.

#### Test-retest repeatability and intraclass correlation coefficient

TRV and ICC of *V*_T_ are shown in Table 3. The TRV of *V*_T_ estimated with 2-TCM was the lowest in the cerebellum (12.4 ± 11.0%) and the highest in the caudate (20.5 ± 9.8%). The mean TRV of *V*_T_ across all brain regions estimated with 1-TCM (15.7%) and Logan GA (16.1%) was similar to that measured with 2-TCM (16.5%). The ICC of *V*_T_ obtained with all three methods of analysis was moderate to low depending on the brain region examined. The ICC of *V*_T_ by 2-TCM was the highest for the cerebellum (0.644) and the lowest for the caudate (0.291). Lower reliability was observed in the arterial input function than brain uptake (Table 4). The *f*_P_ also showed relatively large variability (21.2%) and low ICC.

**Table 3:** Test-retest variability and intraclass correlation coefficient of *V*_T_ by 1-TCM, 2-TCM, and GA

TRV							ICC		
	1-TCM		2-TCM		GA		1-TCM	2-TCM	GA
	Mean (%)	SD (%)	Mean (%)	SD (%)	Mean (%)	SD (%)			
CERCX	11.7	11.3	12.4	11.0	11.5	10.5	0.646	0.644	0.667
CAU	19.2	10.4	20.5	9.8	19.6	12.9	0.261	0.291	0.193
PUT	14.7	9.1	16.1	7.7	15.8	9.3	0.577	0.487	0.509
THA	15.8	9.2	16.6	9.0	16.5	10.6	0.484 0.504	0.425	
LFC	15.8	9.4	17.4	8.1	16.2	10.0	0.505	0.492	0.486
LTC	14.3	8.0	14.7	8.3	14.2	8.2	0.535	0.542	0.507
HIP	15.5	8.7	16.%	7.1	16.4	8.8	0.535	0.548	0.508
LOC	15.0	8.7	15.5	8.9	15.2	8.7	0.568	0.565	0.561
LPC	16.7	9.9	17.6	9.0	17.0	10.8	0.579	0.599	0.573
ACC	15.4	7.3	15.1%	8.8	15.6	7.3	0.487	0.418	0.465
PCC	15.5	9.6	15.8	8.4	15.3	7.9	0.401	0.443	0.412
AMG	18.5	8.5%	19.6	9.3	19.3	9.2	0.454	0.417	0.390

*CERCX*, cerebellum; *CAU*, caudate; *PUT*, putamen; *THA*, thalamus; *LFC*, frontal cortex; *LTC*, temporal cortex; *HIP*, hippocampus; *LOC*, occipital cortex; *LPC*, parietal cortex; *ACC*, anterior cingulate cortex; *PCC*, posterior cingulate cortex; *AMG*, amygdala

**Table 4:** Test-retest variability and intraclass correlation coefficient of area under the curve of GM, input, ratio GM / input, and *f*_P_

	TRV		ICC
	Mean (%)	SD (%)	
GM (AUC)	5.4	3.6	0.857
Input (AUC)	9.8	6.6	0.789
GM/input (AUC)	11.2	11.0	0.638
GM (*V*T)	14.1	8.5	0.584
*f*_P_	21.2	9.5	0.663

*AUC,* area under the curve; *GM*, gray matter

## Discussion

In this study, we evaluated in healthy volunteers a novel PET radioligand for brain imaging of BACE1, [^18^F]PF-06684511. The whole-body biodistribution and dosimetry as well as the quantification and test-retest reliability were examined in two separate studies.

The results of the dosimetry study showed that the calculated ED of [^18^F]PF-06684511 was 25 μSv/MBq, which is similar to the ED of [^18^F]FDG (19 μSv/MBq) and ^18^F-labeled radioligands used for Aβ imaging in AD ([^18^F]AV-45, 19 μSv/MBq; [^18^F]BAY94-9172, 15 μSv/MBq; [^18^F]GE067, 34 μSv/MBq) [11–13]. Contrary to the results obtained in NHPs, there was no evidence of uptake in the gastric lumen, suggesting that the relatively high radiation effective dose (43 μSv/MBq) in the previous NHP study was likely related to experimental conditions or species differences between NHPs and humans [5].

The critical organ receiving the highest dose of [^18^F]PF-06684511 was the pancreas. The high uptake in the pancreas was most likely due to the expression of BACE1 in the exocrine glandular cells [14, 15]. Of note, the uptake of [^18^F]PF-06684511 in the gall bladder and small intestine increased over the whole duration of the study. The pattern of elimination observed for [^18^F]PF-06684511 suggests that the radioactivity is eliminated through the urinary system, and the radioligand and/or its metabolite(s) also undergo enterohepatic recirculation. Based on the calculated effective dose of [^18^F]PF-06684511, the administration of 400 MBq corresponds to a radiation dose of 10 mSv, suggesting that [^18^F]PF-06684511 can be used in clinical trials to measure the brain BACE1 level and/or the occupancy by BACE1 inhibitors in AD.

The results from the test-retest study showed that the distribution pattern of [^18^F]PF-06684511 was similar to the one observed in NHPs, with wide distribution throughout the brain and relatively higher uptake in subcortical regions and relatively lower uptake in the cerebellum [5]. This pattern of distribution is similar to the one previously reported in post-mortem human brain tissue [16].

The quantification of [^18^F]PF-06684511 binding to BACE1 was well described by the 2-TCM, which was the preferred method for the quantification based on the lower AIC and the results of the *F*-test compared with the 1-TCM. Logan GA would be an alternative method considering that *V*_T_ values were well correlated to 2-TCM analysis despite a slight underestimation. These findings suggest that [^18^F]PF-06684511 is a suitable radioligand for imaging BACE1 in the human brain.

The evaluation of the time stability showed a small bias of *V*_T_ (3%) when the duration of the image analysis was reduced to 60 min. Across subjects, the standard deviation of *V*_T_ estimates increased with the reduction of time duration for image analysis and was larger than 5% with 87 min of data. These data suggest that an imaging duration of at least 90 min would be sufficient for the quantification of [^18^F]PF-06684511 binding to BACE1 in the brain. An acquisition protocol of 90 min would therefore be possible in early or prodromal AD patients.

The TRV of *V*_T_ was around 16%, similar to that observed for other radioligands without a reference region such as [^18^F]JNJ-64413739 (12%) [17], [^18^F]PBR111 (16%) [18], or [^11^C]PBR28 (18%) [19], albeit with moderate reliability of the measurements as indicated by the ICC. Considering the measured TRV, it is expected that more than 20% change of *V*_T_ is necessary in clinical trials aimed to measure significant BACE1 inhibition. The variability was larger for the arterial input function than for the brain uptake. Further analysis is needed to assess whether simplified methods, such as SUV or ratio method within the brain regions, can be used in clinical studies in AD patients.

In the test-retest study, two different PET systems, HR (*n* = 5) and HRRT (*n* = 3), were used. The use of PET systems with different resolutions might have affected the estimation of the outcome measures or their reliability. Despite the use of two different PET systems, the mean difference of *V*_T_ between two systems was + 1% (range, − 16 to + 17%) across regions, and the mean TRV was 16.5% (13.5 to 22.6%) and 16.5% (10.4 to 24.1%) for HR and HRRT, respectively. These findings indicate that the use of different PET systems most likely had a minimal effect on the results.

Our dosimetry and test-retest studies indicate that [^18^F]PF-06684511 is a suitable PET radioligand to measure BACE1 levels in humans. Clinical trials using BACE1 inhibitors for AD have been terminated due to safety reasons, suggesting that inhibition of BACE1 activity may not be beneficial for treating AD [20]. [^18^F]PF-06684511 could be useful to assess levels of target engagement associated with undesired effects and further clarify the role of BACE1 in humans. In addition, together with the existing suite of radioligands (e.g., amyloid and tau PET ligands), [^18^F]PF-06684511 could be explored in the evaluation of patients with AD, i.e., quantification of BACE1 expression in the brain and its correlation with disease progression.

## Conclusions

In this study, the BACE1 radioligand [^18^F]PF-06684511 was evaluated for the first time in healthy volunteers. We evaluated the radiation dose, the quantification, and test-retest reliability of the binding [^18^F]PF-06684511 to BACE1. The ED of [^18^F]PF-06684511 was 25 μSv/MBq, which was similar to other ^18^F radioligands. The brain kinetics was well described by 2-TCM, and the TRV of *V*_T_ was around 16%, similar to other radioligands without a reference region. These data indicate that [^18^F]PF-06684511 is a promising PET radioligand to measure the BACE1 level in the human brain.
